# The Impact of Job, Site, and Industry Experience on Worker Health and Safety

**DOI:** 10.3390/safety5010016

**Published:** 2019-03

**Authors:** Emily J. Haas, Brianna Eiter, Cassandra Hoebbel, Margaret E. Ryan

**Affiliations:** National Institute for Occupational Safety and Health, Pittsburgh Mining Research Division, Pittsburgh, PA 15236, United States;

**Keywords:** compliance, hazard recognition, health and safety management system, risk avoidance, self-escape competence

## Abstract

A debate exists about the impact of mineworker experience on health and safety (H&S). Studies often assert that length of time on the job (tenure) is negatively associated with accidents (i.e., new employees have a higher accident rate). However, inferences are all made based on reported incidents, whereas we know that underreporting is a problem in high-risk occupations. To that end, this study sought to examine how worker experience may impact a variety of H&S outcomes on the job. Comprised of three separate case studies with different H&S outcome variables, researchers broke down the results of several data sets that were collected from 3400 miners who worked in either underground coal, surface sand, stone, and gravel, or metal/non-metal to reveal any underlying trends among differing levels of experience on a specific job, with a specific company, and in the mining industry. Each case study is described in turn, using Kruskall-Wallis tests to determine the impact miners’ experience on hazard recognition accuracy (Case 1), self-escape confidence (Case 2), and safety compliance (Case 3). The results show that workers with more job experience possess higher levels of perceived health and safety skills, including the identification of hazards on the job. We discuss the impact of experience on several predictors of incidents, including perceived job knowledge and hazard identification, and perceived compliance on the job. Practitioners can expect to gain a greater understanding of their workforce, including actual differences and similarities to consider, when communicating pieces of their health and safety management system to training workers of all experience levels.

## 1. Introduction

It is common for occupational health and safety (H&S) research to discuss the dynamic environments of high-risk workplaces, including mining, construction, forestry, oil and gas, and transportation. Although the ongoing presence of hazards is real, in some ways, the risks and hazards present in a workplace are fairly static. For example, hazards consistently identified at mine operations include slips, trips, and falls, as well as electrical issues, guarding, or noise and dust exposure [1–3].

Despite a specific hazard or group of hazards being constant in many mine environments, workers often are not. Rather, general occupational research has found that the workforce is constantly changing due to issues such as employee turnover, new people coming in, or job reassignment [4]. These workforce issues are particularly prevalent in industries that have suffered a higher turnover for the past number of years, such as mining, which will continue as baby boomers retire [5]. In response, understanding the knowledge, perceptions, and behaviors of workers, based on any individual differences, may be important for identifying the likelihood of preventing and introducing risks on the job for certain subgroups of workers. In this case, we focus on workers’ experience levels in different job tasks, with their company, and within the industry as individual factors of interest.

To date, even the largest meta-analyses have revealed mixed findings pertaining to the relationship of job tenure and job performance, with more attention being focused on the risks that new employees bring to the job site [6]. Specifically, nearly a century’s worth of research has consistently shown that workers new to their profession are more likely to be injured on the job than those with longer job tenures [7–9]. A commonly referenced landmark study by Leigh and colleagues [8] asserted that length of time on the job (tenure) was negatively associated with accidents (i.e., new employees had a higher accident rate). Similarly, research has also confirmed that job tenure can be positively related to task performance over time [10].

Older studies have shown similar results in coal mining, specifically, where job tenure was found to be a significant factor in accidents that occurred between 1976 and 1977 [11]. These researchers found that 40% of reported injuries had occurred during workers’ first year of employment. More recent research by Groves and colleagues [12] used data from the Mine Safety and Health Administration (MSHA) between 1995 and 2004 to conclude that 28% of injuries and 31% of fatalities occurred among employees who were in their first year on the job. MSHA is the regulatory entity for U.S. mining, mandating specific Codes of Federal Regulations (CFRs) which organizations must follow. Similarly, Kecojevic and colleagues [13] analyzed the relationship between work-related fatalities and worker experience to find that 44% of workers killed in equipment-related incidents had less than five years of mining experience.

Although these numbers seem convincing, there are other factors to consider. First, the statistics can be recontextualized. For example, Kecojevic’s results [13] could communicate that 56% of those workers killed in equipment-related incidents had more than five years of mining experience, indicating that this more experienced population is overlooked when considering specific risk management interventions. Specifically, many studies do not examine the spectrum of the workforce and often focus on workers who tend to be inexperienced (e.g., under one year on the job). One research study showed a strong inverse relationship between job tenure and injury claim rates, but job tenure only ranged from 0 to 13 months [14]. Importantly, these inferences are all made based on reported incidents, whereas we know that underreporting is a problem in high-risk occupations [15]. In light of these issues, a more diverse research portfolio that includes all levels of experience, from novice to veteran, is needed to better understand this phenomenon.

As this review demonstrates, there is still much disagreement on the impact of workers’ demographic factors, specifically experience, on performance outcomes. Therefore, more recent and even self-reported data that offer the ability to study a variety of experience factors, as well as to determine accurate occurrences of incidents, are necessary to uncover any trends in potential indicators of incidents, if they exist. To that end, the National Institute for Occupational Safety and Health (NIOSH) researchers analyzed several quantitative datasets that were collected by working with miners who worked in either underground coal, surface stone, sand, and gravel, or metal/nonmetal to reveal any underlying trends among differing levels of experience. These case studies include several sectors of the mining industry. The mining industry remains an important part of economies both domestically and internationally. Regardless of the sector (e.g., coal, metals, minerals, etc.) and whether materials are extracted on the surface or underground, the working conditions are consistently acknowledged as physically demanding, and workers are exposed to numerous risks [16]. In 2007, National Public Radio [17] interviewed mineworkers to better understand their job and work environment, and a common theme among employees was that the work is “dark, dirty, and dangerous.” Due to the numerous hazards present both underground and on the surface, research to understand ways to improve the H&S workers through protective technologies and evolving risk management processes remains important.

Mineworkers completed respective surveys over a two-year period between 2016 and 2018. These data, collected in three separate case studies, used time in job, time at the mine, and time in the mining industry as predictor variables in an attempt to find associations with workers’ perceived knowledge, confidence, and job compliance. These specific case studies provide results that show the necessity of understanding how experience, on multiple levels, can impact worker perceptions and performance in order to improve hazard recognition, compliance to safety rules, and if necessary, rapid response in the event of an emergency. Although the results in this study are specific to mining, the trends are still relevant to any high-risk industry to help develop and implement aspects of a tailored risk management program to segments of the workforce at opportune time periods. Whether focusing on the development and implementation of more accurate hazard recognition and training, adequate preparation and effective response during a mine emergency, or improved safety climate through targeted leadership and engagement in H&S, all are efforts that can benefit from more information about the workforce at varying points in their careers.

## 2. Applied Research Methodology

Prior to the start of each respective data collection, NIOSH researchers obtained Institutional Review Board (IRB) and Office of Management and Budget (OMB) approval. In total, 3428 surveys were completed with mineworkers over a two-year period. These data, collected in three separate case studies, use time in job, time at the mine, and industry tenure as predictor variables in an attempt to find associations between workers’ varying experience and performance outcomes of interest (i.e., hazard recognition, self-escape confidence, and compliant behavior). Each of these case studies are presented in turn and utilize similar analysis methods to convey the results related to experience. Regarding how experience was measured within each case study, the categories measured were under one year, 1–5 years, 6–10 years, 11–15 years, 16–20 years, and more than 20 years. These categories are consistent with the demographic breakdown included in NIOSH’s Quality of Worklife Questionnaire and General Social Survey. These surveys have generated reports since 1972, exhibiting reliability and consistency in demographic breakdowns. Additionally, MSHA asserts that a novice worker has under one year of experience, further supporting our justification for the inexperienced group.

Prior to running analyses within each case study, an assessment of the normality of the data was completed using either a visual inspection (i.e., Case Study 1) or the Kolmogorov-Smirnov test (i.e., Case Studies 2 and 3). For Case Studies 2 and 3, the Kolmogorov-Smirnov test rendered a result of less than 0.05, showing a deviation from normal distribution. This is normal and even common for large datasets [18]. In response, a non-parametric option, the Kruskall-Wallis, was used as an alternative to a one-way between groups ANOVA for all three case studies. Similarly, in order to understand exactly where each significant relationship existed, Mann-Whitney tests were completed for each group to further investigate differences between the various experience groups and outcomes of interest. To better account for a potentially inflated type I error, due to multiple testing, the researchers adopted a more stringent significance value of 0.01 as an alternative to Bonferonni tests for each potential relationship. Further, we report the skewness and Shapiro-Wilks test of normality to demonstrate the non-normal distribution of the results, further justifying the use of nonparametric tests.

### 2.1. Case Study 1: Time on the Job and Hazard Recognition Accuracy

The first case study reflects on whether mineworkers’ time in their current position affects their ability to accurately recognize worksite hazards. Hazard recognition is fundamental to every safety activity, and hazards that go unrecognized and unmanaged can potentially result in catastrophic accidents and injuries [19]. This is especially true for the mining industry because the environment is dynamic and often unpredictable, and mineworkers perform a variety of tasks in close proximity to heavy machinery [20]. Despite the importance of hazard recognition, recent research indicates that a large proportion of hazards go undetected by mineworkers [1,21].

While previous research has mainly focused on determining the effect of overall tenure within an industry on hazard recognition ability [1,22], other types of experience are also critical. As an example, the specific job a worker does at a mine site has been shown to affect the number of hazards found during a hazard identification task, with exploration workers finding a greater number of hazards than those who perform maintenance jobs [21]. This difference is presumably because workers in an exploration role are exposed to more and varied areas at the mine. An alternative way to capture the impact of this type of experience is to look at the effect of time in current position on hazard recognition ability. As an industry, mining has experienced periods of turnover, because of retirement or layoffs, and as a consequence, the overall industry demographics have changed (e.g., [5,23]). At the mine site level, this has caused workers to change job positions. Therefore, the purpose of this case study was to identify the influence of time in current position on hazard recognition ability.

#### 2.1.1. Case Study 1 Materials and Methods

After researchers obtained IRB and OMB approval, study participants were recruited to travel to the NIOSH Pittsburgh Mining Research Division in Bruceton, PA, where the study took place at the facility’s Virtual Immersion and Simulation Laboratory (hereafter referred to as the VISLab).

##### VISLab and Panormic Images for Hazard Identification.

The VISLab contained a 360° panoramic projection screen that measured 10 m in diameter by 3 m tall. Imagery was front-projected onto this screen from six high-definition projectors to create a seamless image. For the purposes of this study, the VISLab was equipped with 10 motion-tracking cameras and used in conjunction with eye-tracking glasses to record participant movement and resolve their point of regard within the display space, and study image sequencing was controlled by an in-house application. Research materials for the laboratory case study included 32 panoramic images of four locations typically found at any surface stone operation: pit, plant, roadway, and shop. These are all plant location areas that are included in the planning processes of controlling major hazards at surface operations, followed by identifying material unwanted events (MUEs), which then leads to the critical control management process [24].

Eight panoramic images were taken at each of the four mine-specific locations. Six images for each of the four locations were experimental images. These 24 experimental images contained hazards that were staged by the researchers based on common citations and incidents recorded in MSHA’s 2015 database [25]. Two images for each of the four locations, totaling eight images, were control images that contained no hazards. The number of hazards per experimental image ranged from two to seven, totaling 101 hazards among the 24 images. The overall breakdown of the hazards was 19 in the pit, 25 at the plant, 26 on the roadways, and 31 in the shop. Hazards varied by type, size, location, and risk. Researchers did not focus specifically on one type of hazard (e.g., slips, trips, or falls, or electrical). Instead, we included hazards that were appropriate given the context and location. Choosing to create scenes this way means that some hazard types included several representations, while other hazard types included one or two representations. This approach makes it difficult to compare hazard groups, but that was not the primary goal. The goal was to create panoramic images that were realistic and accurately reflected hazards and situations that mineworkers encounter during their workday. Other publications provide a more detailed explanation of the panoramic images and the hazards within them (e.g., [1,26]).

##### Participants.

Participants consisted of mine H&S professionals, mineworkers, and students enrolled in a mining engineering program. All mine H&S professionals and mineworkers had experience with surface stone, sand, and gravel mining operations and procedures and reported having completed at least 24 h of MSHA New Miner Training and those with more than one year of mining experience reported having completed the additional eight hours of MSHA Annual Refresher Training as necessary. These trainings are mandated per MSHA’s Code of Federal Regulations (30 CFR Part 46) [27].

Fifty-two participants volunteered to take part in the study. However, three participants were excluded from the analyses because of technical difficulties; therefore, the final dataset included 49 participants. All participants had normal or corrected to normal vision and were screened to verify their visual abilities. None of these participants received payment for their participation, although some were able to participate during a normal workday with permission from their supervisors. Of the 49 participants, 19 (39%) were 18–24 years old; 12 (24%) were 25–34 years old; 6 (12%) were 35–44 years old; 9 (18%) were 45–54 years old; and 3 (6%) were 55–64 years old. Participants were also asked to report time in their current job and total time in the mining industry. Because of the small sample size, the participants initially included in the 16–20 year job experience group were combined with the 11–15 year group because their time in position was similar and provided the opportunity for enhanced statistical analyses (see Table 1).

##### Procedure.

After researchers obtained informed consent, participants were outfitted with eye-tracking glasses that were connected to a small laptop placed in a backpack, also worn by the participant. Participants were given a handheld joystick in their dominant hand that was connected to wireless data streaming hardware that was placed in the backpack. The eye-tracking glasses had passive motion-tracking markers to track head position, and several additional markers were placed on the participant’s torso to resolve head motion relative to the body frame. A series of calibration tests for the motion-tracking system and eye-tracking glasses was conducted in the simulator to ensure that the data collection software was accurately capturing the participant’s gaze within the screen space. Once the data collection instruments were calibrated, researchers presented two panoramic images to the participants to allow them to familiarize themselves with the 360° simulator and button press control.

Once acclimated, participants were presented with the 32 images in four sets of eight grouped by location category (pit, plant, roadway, and shop). Blocks were randomized across participant categories, and images were randomized within each location category. Participants were given up to two minutes to view each image and were instructed to press the joystick button as quickly as possible when they identified a hazard. If they decided they had identified all the hazards in an image, participants could press a second button on the hand grip to end that trial early. Once all images were complete, the glasses, markers, and backpack were removed.

#### 2.1.2. Case Study 1 Results

**Figure 1.**
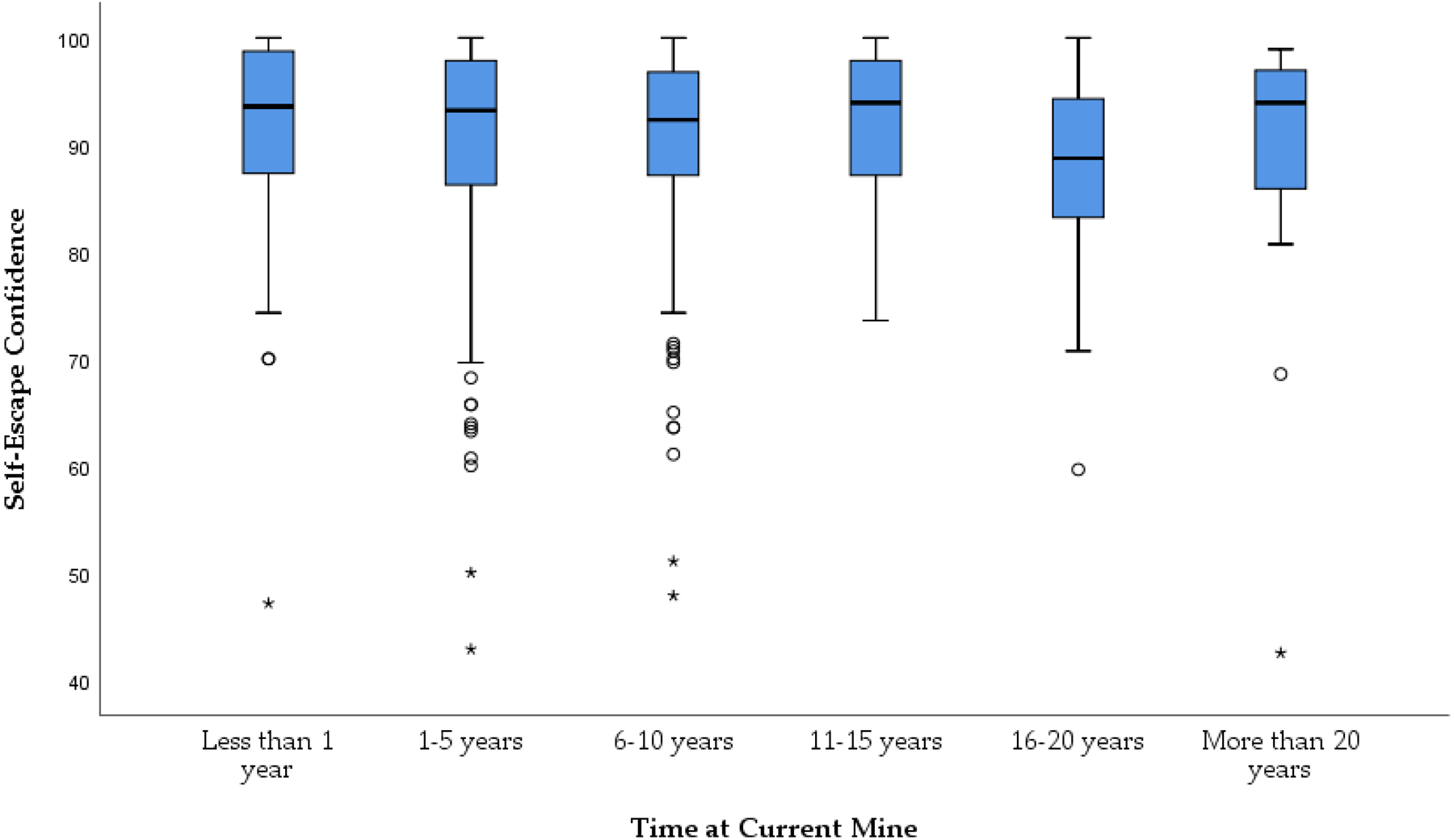
A box plot depicting the distribution of average confidence of hourly workers across 28 self-escape competency items (on a 100-point confidence scale) based on time at current mine. The circles are outliers and the asterisks are extreme outliers. About 95% of the data falls between the inner fences (i.e., the t-bars that extend from the boxes).

The Kruskal-Wallis Test was applied to the data to determine the effect of time in position on hazard recognition accuracy. The raw averages are presented in Table 1, while Figure 1 visually shows the difference in hazard recognition accuracy and job experience. As the results visually show, the hazard recognition accuracy was lowest for those with under one year of experience in their current job position. Recognition accuracy gradually increased until workers exceeded 20 years in their same job role; at this point, the recognition accuracy dropped significantly.

The Kruskal-Wallis Test revealed a significant difference in hazard recognition accuracy scores across the experience groups, χ^2^ (5, *n* = 49) = 11.832, *p* = 0.01. To further identify where these relationships existed, Mann-Whitney tests were completed between each of the experience groups. Of the possible comparisons, one was statistically significant, using 0.01 as a strict significance value to account for possible inflated Type I errors (see Appendix A for significant relationship). The Mann-Whitney tests indicated that the group with less than one year of experience in their current position found significantly fewer hazards than all other groups in their current position. Obviously, a majority of the participants are in the low experience amount groups. Even though the distribution is uneven, the results are consistent with previous research which shows that increasing experience improves hazard recognition (highlighted in the discussion section of this paper).

### 2.2. Case Study 2: Time at Current Mine and Confidence in Risk Response

The second case study reflects on underground coal miners’ tenure at their current mine and applies this variable as a potential indicator of mineworkers’ self-escape competence. Because mine disasters are high-severity, low-probability events, there are limited data related to post-disaster survival of underground miners. Although these events are fortunately rare, preparedness deficiencies often come to light when they occur. Even if a miner is fortunate enough to survive an initial catastrophic event (e.g., explosion, gas or water inundation, roof fall), he or she may still be required to successfully execute critical physical and cognitive tasks to escape from the mine unaided.

Limited research into such circumstances suggests that a lack of competence in the non-routine tasks required of mineworkers during emergency situations can have tragic consequences (e.g., [28–30]). While significant efforts to improve mine emergency responses have been made in the last decade, it is impossible to know with any certainty whether they have been effective or whether active underground coal miners possess the knowledge and skills required for successful self-escape. Therefore, after researchers identified critical tasks and required skills through detailed task analyses (see [31,32]), the question of mineworker competence was addressed.

#### 2.2.1. Case Study 2 Materials and Methods

Because it is difficult and dangerous to simulate the dynamic conditions of an actual mine emergency and because standard self-escape competency and assessment protocols are yet to be developed, it was necessary for researchers to frame the questions in a way that gaps in competence could most readily be identified and quantified. A large body of research suggests that when competence is difficult or impossible to measure, self-reported confidence in one’s ability to perform a task can serve as a reliable “proxy” measurement of competence [33,34].

##### Self-escape Competence Survey.

This survey, administered by NIOSH, consisted of questions that measured mineworker self-escape competence. To identify gaps in the critical self-escape knowledge and skills among mineworkers, 28 critical self-escape tasks that were identified in previous NIOSH research and preliminary task analysis results [31,32] were phrased into confidence questions, which were subsequently reviewed by mine emergency response subject matter experts for content validity. The 28 items were deemed to be skills that all miners, regardless of the self-escape role, should be able to confidently demonstrate or explain in the event of a mine emergency [35]. Participating mineworkers were asked, “On a scale of 0–100%, how confident are you that you could correctly demonstrate or explain the following to a brand new miner?”

Table 2 lists the items that participants responded to using an 11-point scale that ranged from not at all confident to extremely confident [36]. When doing an internal reliability test of these 28 variables, the Cronbach’s *α* = 0.96, demonstrating high internal consistency. Additionally, the inter-item correlations are strong, which further justifies using average confidence as a summary variable in this case study. Questions also captured demographic data including age, time in mining, time in job, and time in current mine, and responses to other background questions related to leadership experience, specialized training, and emergency response experience.

##### Procedure.

Upon receiving IRB and OMB approval, NIOSH researchers invited underground coal mine operations across the United States to participate in the survey using a variety of convenience and purposive sampling methods [37]. Although the hope was to visit mines in all geographic regions, interest in the survey came primarily from mine operators in the Eastern U.S. and, as a result, all data were collected in the Appalachian Region.

##### Participants.

Researchers traveled to the eight mines and administered a paper-pencil survey. Potential participants were instructed that the survey was not only voluntary, but also completely confidential and that their responses would not be shared with their supervisors, except in aggregate form. On average, the survey took about 10 min to complete. Eight mines participated in this data collection effort in 2016 and 2017, with 696 hourly workers completing the survey (note, salaried workers also participated, but are not included in this analysis because they are not likely to have to lead or be able to escape during an underground emergency). Of the 696 hourly participants at the eight mines, the range of participants per mine was 16 to 213 (M = 87).

#### 2.2.2. Case Study 2 Results

**Figure 2.**
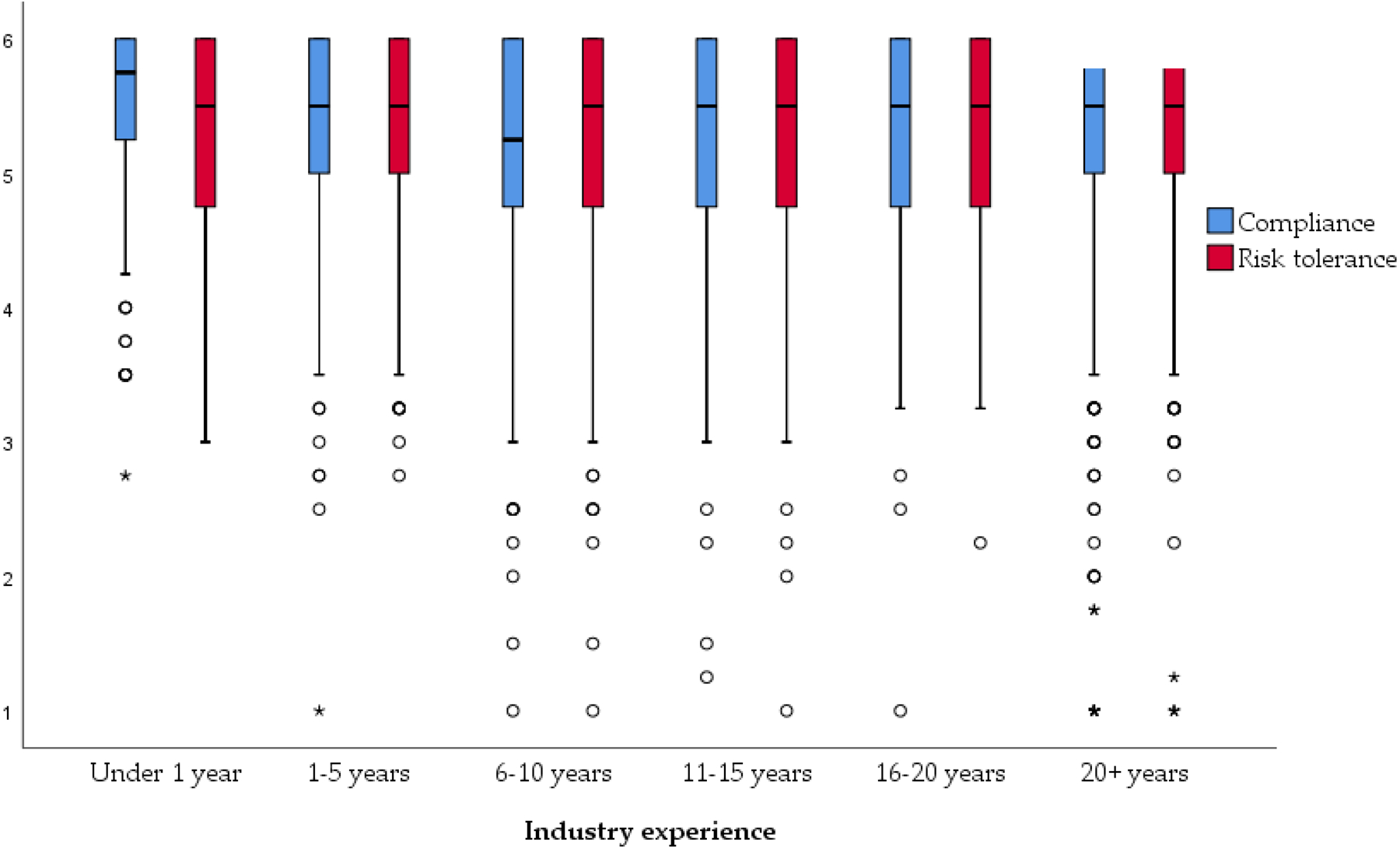
A box plot depicting the distribution of average compliance and risk avoidance among workers based on mining experience. The circles are outliers and the asterisks are extreme outliers. About 95% of the data falls between the inner fences (i.e., the t-bars that extend from the boxes).

For this analysis, the main outcome variable of interest was self-escape confidence. This variable is an average of each individual respondent’s self-reported confidence on each of the 28 self-escape competencies included on the survey. Because the variable is highly skewed (skewness = −1.81, standard error = 0.10) and not normally distributed (Shapiro-Wilks statistic = 0.84, *p* = 0.000), researchers used nonparametric tests to measure associations between self-escape confidence and experience. The self-escape confidence median and range within each experience group are shown in Table 3. Figure 2 plots the distribution of the self-escape confidence variable by level of experience.

As illustrated by Figure 1, the self-escape confidence variable is highly skewed to the left for each level of worker experience, with self-reported confidence more likely to be on the higher end of the spectrum. Figure 2 also highlights the presence of outliers, which might allude to a more nuanced association between worker experience and self-escape confidence that might be clearer when taking other individual factors into account (e.g., age, education, workgroup or experience in emergency response, etc.).

The Kruskal-Wallis Tests for each of the 28 competence questions revealed that six were statistically significant across the experience groups. Table 4 shows these items and their respective results. In order to understand where exactly these significant relationships existed, Mann-Whitney tests were completed between each group for each item to further investigate differences between experience groups. In completing these tests for each possible comparison group, 32 were statistically significant, using 0.01 as a strict significance value to account for possible inflated Type I errors (see Appendix B).

These results indicate that time at one’s current mine can significantly impact workers’ self-reported confidence on several self-escape competency items. Also of interest is that almost all of the items that rendered statistical significance were mine-specific competencies, such as where to find self-escape self-rescuers (SCSR), how to read a mine map, and knowing the mine’s emergency response plan. Alternatively, some of the items that did not render statistical significance were more mining-specific rather than site-specific, such as how to don (or deploy) an SCSR, how to use nonverbal communication, or how to identify the explosive atmosphere with a gas meter. Additionally, these items of significance are often covered in mine annual refresher trainings.

The trend in these results was somewhat unexpected. Specifically, the results showed that self-confidence was, in most cases, stronger among those workers who were newer to the mine site. Then, worker confidence tends to decrease over time, often hitting the lowest point around 16–20 years. Then, the confidence for those who were employed on site for more than 20 years often started to increase again. These initial results speak to the potential impact of site-specific training for new mineworkers. Specifically, most mineworkers must go through site-specific training upon starting at a new jobsite. These results lend themselves to supporting the initial value of any on-the-job training, communication, or experience that mineworkers gain early on at the mine. However, the results also illustrate that some of these knowledge and skill-building efforts may wane over time as workers’ confidence levels in performing these critical self-escape activities decrease.

### 2.3. Case Study 3: Time in the Mining Industry, Safety Compliance, and Risk Avoidance

The last case study examines time in the mining industry and workers’ perceived compliance to following rules and avoiding risks. In this particular case, a survey dataset was used that was populated by NIOSH researchers who administered a safety climate survey to the industry. Safety climate has been linked to many safety-related outcomes [38–40]. However, as Clarke [38] and others have pointed out, the links have not been consistent across all safety climate research. In response to overarching questions about safety climate and worker performance, NIOSH developed a safety climate survey for the mining industry. Through an extensive literature review of safety climate assessments in other high-risk occupational industries, perception-based organizational value and characteristic constructs were identified and presumed to be important in fostering H&S knowledge, motivation, behaviors, and outcomes.

#### 2.3.1. Case Study 3 Materials and Methods

As a part of the safety climate assessment completed by participants, compliance was one factor, or scale, contained within the survey, as well as risk avoidance. These data were used to answer whether there is a difference in compliance and risk avoidance across experience, or tenure, in the mining industry.

##### Compliance Survey Scale.

Safety compliance is related to workers’ participation in safety-related activities, including the completion of work in a safe manner [41]. More specifically, Griffin and Neal [42] contend that safety compliance is a function of knowledge, skills, and motivation to comply with safety policies and processes. A safety compliance scale was adapted from Neal and colleagues [43] and Zachataros and colleagues [44] to measure compliance with safety procedures. The original scale had a Cronbach’s *α* = 0.94. In the current survey, the scale was adapted to a four-item measure which workers were asked to complete using a six-point Likert scale (strongly disagree to strongly agree), with six being the highest value, indicating a high level of compliance. Our shortened, four-scale version had Cronbach’s *α* = 0.85, demonstrating high internal reliability [44,45]. The four questions were prefaced with “When I’m at work I…” and were phrased as follows:

- don’t take risks that could result in an accident;
- use all necessary H/S equipment to do my job;
- use the correct H/S procedures for carrying out my job;
- always report all health/safety-related incidents.

##### Risk Avoidance Survey Scale.

Measuring risk avoidance can help predict the types of at-risk behaviors in which workers are willing to participate [46] and as a result are used to measure an individual’s general tendency to take risks and general avoidance of risks on site [47,48]. Researchers adapted items from Meertens and Lion’s [48] risk propensity scale. The original scale contained nine items to tap into difference aspects of risk-taking and yielded a Cronbach’s *α* = 0.80. We adapted four of these items and used a six-point Likert scale (strongly disagree to strongly agree), with six being the highest value, indicating a high avoidance of risks. Within the current sample, these questions rendered a Cronbach’s *α* = 0.72, which is an acceptable level of internal consistency [44,45]. Participants were prompted with, “As far as day to day work…”, and then answered the following items:

- Safety comes first;
- I do not take risks with my safety or health;
- I prefer to avoid risks;
- I take risks regularly (reverse-scored item).

##### Procedure.

After IRB and OMB approval, the survey was validated [49] and data collection occurred between February 2016 and March 2018. Upon contacting or being contacted by a corporate H&S leader, mine operator, or H&S manager and explaining the study, a mutually agreed-upon time was chosen to travel to the mine and administer the survey. If upcoming MSHA annual refresher training was scheduled, researchers often visited the mine that day in order to have everyone together at one time. If upcoming annual refresher or other training was not on the mine’s schedule in the near future, researchers worked with the mine to pick one or two days that were convenient to attend pre-shift safety meetings to collect the survey data.

Prior to participating, mine management and hourly workers were briefed about the purpose of the survey, that their participation was voluntary, that their responses would be anonymous, and that their answers would not be seen by their supervisors. To our knowledge, no one refused to participate and it took approximately 15 min for participants to complete the survey. Researchers collected the hard copy surveys and subsequently, they were entered into an SPSS file for cleaning and analysis.

##### Participants.

Participants consisted of 2683 mineworkers—both salaried and hourly—at 39 mine sites. Three of these mines were in Canada and the remaining 36 were dispersed throughout 17 states in the United States. The 39 mines represented nine major companies and three mined sectors (i.e., coal, stone, sand, and gravel, and industrial minerals). To our knowledge, everyone who was present on site during data collection completed the survey. The breakdown of participation by sector was stone, sand, and gravel (*n* = 1418, 53%); industrial minerals (*n* = 907, 34%); and coal (*n* = 358, 13%). The range of participants at each mine was 7–280 (M = 69). Additionally, *n* = 569 (22%) of the participants were salaried workers and *n* = 2020 (78%) were hourly workers.

#### 2.3.2. Case Study 3 Results

For this analysis, there were two main outcome variables of interest: safety compliance and risk avoidance. Because these variables are highly skewed (skewness = −1.63, standard error = 0.048 and skewness = 1.38, standard error = 0.048, respectively) and not normally distributed (Shapiro-Wilk statistic = 0.82, *p* = 0.000 and 0.85, *p* = 0.000, respectively), researchers used nonparametric tests to measure associations between the outcome variables and industry experience.

The median and range of participants’ responses on the compliance scale and risk avoidance scale within each experience group are presented in Table 5, while Figure 2 plots these averages on a line graph for a visual comparison of the patterns by experience groups.

A Kruskal-Wallis Test revealed a statistically significant difference in compliance across the six different experience groups, also shown in Table 6 (Under 1 year, *n* = 234; 1–5 years, *n* = 465; 6–10 years, *n* = 441; 11–15 years, *n* = 389; 16–20 years, *n* = 239; 20+ years, *n* = 761), χ^2^ (5, *n* = 2529) = 32.002, *p* < 0.000. The less and more experienced groups recorded higher mean rank scores than the middle-range experience groups, particularly in the 6–10, 11–15, and 16–20 year ranges. A trend toward significant differences in risk avoidance across the six different experience groups was rendered (Under 1 year, *n* = 239; 1–5 years, *n* = 468; 6–10 years, *n* = 444; 11–15 years, *n* = 395; 16–20 years, *n* = 245; 20+ years, *n* = 770), χ^2^ (5, *n* = 2561) = 10.551, *p* = 0.061), but does not meet the significance requirements established for this study, so detailed results are not shown.

These results indicate that experience in the mining industry does significantly impact workers’ compliant behaviors on the job. In completing these tests for each possible comparison group, several were statistically significant for compliance (see Appendix C for results). Because risk avoidance was not statistically significant, Mann-Whitney tests were not completed for these between-group relationships.

The Mann-Whitney tests show that the group with under one year of mining experience had significantly higher levels of compliance compared to each experience comparison group. In addition, the group that had 1–5 years of mining experience had significantly higher levels of compliance than those with 6–10 and 16–20 years of experience. However, the experience group with more than 20 years in the industry did have higher levels of compliance than one experience group—the 6–10 year group. Most noticeably and notably within these results is that the group with under one year of experience in the mining industry perceives that they follow the rules, report incidents, and use the correct procedures to complete their jobs.

## 3. Discussion

Now that all three of the case studies and their respective results have been explained, it is possible to compare the results across cases to determine trends among various types of experience. The case studies, each presented separately, have slightly different variables, but render some similar and deviating trends that are worth noting. Specifically, these trends can not only be used to inform and improve aspects of an occupational risk management system, but also to tailor practices, activities, and resources for at-risk worker segments in the industry to reduce incidents for workers of varying experience levels. If the results reveal anything, it is first, that there are several types of experience that researchers, practitioners, and management must account for when determining how to improve the execution of specific risk management processes in the workplace. More specific implications are discussed below.

### 3.1. Implications for New Job Task Training and Skill Maintenance

As time on the job increases, studies suggest that workers who experience more near miss incidents eventually have the ability to proactively perceive and identify risks [50]. Similarly, previous research has shown that job tenure is associated with greater job performance because, over time, workers gain more tacit knowledge and can more effectively perform their jobs [10]. The results for case study 1 support previous findings in that hazard identification increased as participants’ job experience increased. These results are not particularly surprising until the job experience group of more than 20 years significantly drops in their ability to detect hazards. There could be several reasons for this drop in recognition ability, including age and decreased senses, such as vision and hearing. Another reason for this drop in hazard recognition may be that, even if individuals with more experience on the job are still performing that task, their role or recent experience in dealing with a hazard may have changed. For example, research in the mining industry has shown that a lack of recent experience on the job or in the environment can impact workers’ abilities to recognize and respond to hazards [51]. Gaps in “recent experience” can occur from short-term layoffs or idle mines, which have been more common in recent years.

It is also possible that workers with more job experience eventually transition into a mentor role and teach job tasks rather than perform them. In this role, these more experienced workers might not be in the position to “see” as many hazards. If this is true, then these results for both those with less and more experience on the job may be interdependent. In other words, if those mineworkers who have decades of experience have competing roles and responsibilities on the job, their recognition of hazard identification may be affected and as a result, they may not pass along critical information to new mineworkers in a specific job task. Therefore, it is important to ensure that all veteran mineworkers who are certified to task train have the ability to accurately and efficiently identify site-specific hazards themselves and that these types of hazards are included in any task training or new, on-the-job training received by new workers. Additionally, ensuring that new workers are exposed to low-hazard conditions at first is necessary, as well as ongoing monitoring of potential hazard exposures [52].

### 3.2. Implications in Risk Management to Maintain Worker Confidence, Compliance, and Risk Avoidance Throughout Tenure

An unexpected trend presented itself in the latter two case studies, showing that workers new to a specific mine or to the mining industry (albeit not controlling for job tenure) tend to be more risk-avoidant, confident in their self-escape abilities, and compliant on the job. These are all potential predictors of overall job performance [41]. Within Case Study 2, workers became less confident in their ability to complete self-escape tasks and reached their lowest levels of confidence after around 16–20 years of time at their mine. It is possible that mineworker self-confidence is negatively impacted through workers’ developing a higher tolerance for risk and becoming less compliant as their time in the industry increases (Case Study 3 showed that mineworkers with 6–10 years of industry experience had the highest levels of non-compliance and risk tolerance). These behaviors went up slightly, but still remained low until workers reached 20 years of industry experience.

These complementary findings are interesting because most organizations focus on resources for newer workers [53,54]. Specifically, much of the safety training developed for new workers is designed with the intent that the main causes of their injuries are attitudes and behavior [54,55]. In other words, much training for new workers might focus on declarative knowledge, known as the facts, rules, and principles on site [56]. However, our current results show that a gap in procedural knowledge may be occurring on mine sites. Procedural knowledge is the “application of declarative knowledge in practice” [6] (p. 307) and appears to diminish as time at a location and with the industry increases. In other words, as experience on site and within the industry increases, workers may rate certain hazards and risks to be less salient.

Although our results seem surprising (e.g., workers become less confident and less compliant over time), other research in occupational H&S has shown that extended length on the job at the same workplace significantly decreases workers’ intrinsic motivation, while increasing their feelings of boredom and dissatisfaction [57]. In addition, the literature suggests that longer tenure contributes to workers’ perceptions of their jobs having low task variety, which in turn leads to lower motivation to perform their core tasks diligently [57,58]. In other words, workers may be more likely to fall into a maintenance mode on the job versus a learning mode. Another term for this is risk normalization and is discussed by researchers as a contributor to rating the severity of certain risks as lower than they used to early in their careers [59]. These risks seem to appear normal over time and as a result, workers are more likely to take risks [60]. In mining, however, this phenomenon has not been explored too often, most likely because other studies have shown that if workers are dissatisfied with their job after about five years, they tend to move on to a different career path, workplace, or job task [61].

Notably, the mining industry employs a distinctive segment of the working population. Specifically, based on geography, the closest mine may be the highest-paying or only job available to many people in rural areas. As a result, it is possible that if workers are bored or dissatisfied on the job, a lack of resources and opportunities could prevent them from pursuing other opportunities. The statistically significant drop in worker compliance once workers hit six years in the industry supports this previous research and underscores the importance of mine organizations helping workers to stay engaged and maintaining their procedural knowledge on a daily basis. Making subtle improvements to an organization’s risk management processes and communication could positively impact workers’ perceptions of the organization, as well as their commitment to the company [6].

### 3.3. Changes to Risk Management Practices

In recent years, it has become accepted that factors contributing to occupational H&S incidents are related to the organizational risk management practices that are in place within the work environment [62]. Specifically, attention to the varying programs and processes in place is critical to preventing individual accidents from occurring and reoccurring [63,64]. For example, Pietilä and colleagues [65] determined that the probability of incident reoccurrence is substantial after one’s first injury and that more effective prevention measures should be put in place on a routine basis.

Our results suggest that organizations should strive to make changes to better target, engage, and involve workers so that they can maintain high levels of compliance and risk awareness throughout their careers. Some research suggests that redesigning jobs for workers can improve their motivation and organizational commitment (e.g., [57]). More recent results from a compilation of meta-analyses [6] suggest the need for job rotation, even if vertical options are not an option, because workers can learn something new and be reengaged on the job [58]. This is particularly important when workers need to know certain competencies to escape during a mine emergency. In this case, job rotations could be especially useful for mineworkers to reengage with learning specific aspects of their mine that would assist their self-escape if the conditions suddenly degraded.

Importantly, making changes to existing organizational risk management systems and improving leadership mechanisms are likely to be difficult and will take buy-in from the top before trickling down to the worker level. In the interim, flexible leadership approaches are critical for not only effective risk management, but also specific emergency management approaches in place on site [66]. Wise [66] also emphasized the value of adaptive management for adaptive workplaces. These efforts focus on increased communication and collaboration to support organizational learning and make space for any quick reactions needed by workers. Therefore, managers fostering an environment where visibility and teamwork are apparent could help maintain both the declarative and procedural knowledge needed to maintain safety and health on the job. Researchers and practitioners argue that strong leadership and communication can help mitigate normalization of deviance on site [60].

## 4. Conclusions and Limitations

It is an obvious limitation that we are drawing upon patterns using three varying datasets that are all based on behavioral lab or self-reported data. If researchers were able to coordinate data collections and specific instruments to a more rigorous degree, stronger relationships and results may have emerged. For example, being able to track the same groups of people to compare how they perceive and process risks that stem from routine and non-routine work practices, would be an area of future exploration that could occur with a common sample. However, the results for each separate case study all support findings in previous studies, supporting the validity of all three studies and their potential influence on future organizational research. In our case, synthesizing the results of the three studies together allowed for some sort of triangulation, particularly among the second and third case studies, which provide support for increased communication and on-the-job experience and self-assessments that can support the ongoing development and maintenance of procedural knowledge. However, future research should aim to better coordinate big data collection efforts with overlapping populations in order to draw more cause-effect results. In addition, such data should be linked to lagging indicator data [67] to better support the conclusions made throughout this paper. The current case studies did not collect accident or fatality data so these concrete links cannot be made.

Despite all the limitations, this paper was able to contribute to the occupational H&S literature in several ways. First, we were able to show that organizations need to consider worker factors other than age and blanket experience as possible predictors of worker performance. The main takeaway is that even if longer job tenure is associated with safer job performance, as shown in Case Study 1, this may not be the case as workers’ tenure increases at the mine site and within the industry as an overall career path. More specifically, our results demonstrated that communication with more intermediate to veteran workers (i.e., 6–20 years) is necessary to maintain worker engagement and execution of the organization’s health, safety, and risk practices.

In summary, these three case studies show the value of being able to identify various levels of workers’ experience, and that it is important for an organization to continually reengage with workers about specific safety issues relevant to their job tasks, at their mine, and within their mining sector. In doing so, managers may be able to parse out important differences in their workforce’s knowledge, attitudes, and behaviors based on varying levels of experience, in order to reduce negative consequences for managers and their organization while improving the knowledge, motivation, and overall resources for the workforce.

## Figures and Tables

**Table 1.** Hazard recognition accuracy by time in current job position.

Time in Position	% Sample	Hazard Recognition Accuracy
Under 1 Year	47%	49%
1–5 Years	31%	58%
6–10 Years	8%	59%
11–20 Years	8%	65%
20+ Years	6%	56%

**Table 2.** Self-escape competency items contained in case study 2 survey.

How to don a self-contained self-rescuer (SCSR) When to don an SCSR How to use nonverbal communication How to use tetherline How to identify explosive atmosphere with gas meter Escapeway locations How to test roof conditions Where to report in event of emergency What to expect when wearing an SCSR When to enter a refuge alternative (RA) RA locations Tetherline locations SCSR cache locations Escapeway map locations	How to fight a fire Lifeline symbols When to fight a fire How to operate RA When to construct a barricade Communication and tracking system Own role in mine’s emergency response plan (ERP) How to read mine map symbols What alarms/alerts mean Ventilation/smoke leakage How to reestablish ventilation How to construct a barricade Chain of command for reporting emergency Mine’s emergency response plan

**Table 3.** Self-escape confidence across 28 competency items by time at mine (*n* = 696).

Experience	% Sample	Median	Range
Under 1 Year	13%	93.6	52.9
1–5 Years	32%	93.2	57.1
6–10 Years	37%	92.3	52.1
11–15 Years	11%	93.9	26.4
16–20 Years	4%	88.8	40.4
20+ Years	3%	93.9	56.4

**Table 4.** Kruskal-Wallis results for six self-escape competency items (only significant differences included).

Scale	Experience	*N*	Mean Rank	Chi-Square	df	Asymp.Sig
What to expect when wearing an SCSR	<1 year	88	409	21.2	5	0.001
	1–5 Years	212	351			
	6–10 Years	254	335			
	11–15 Years	85	325			
	16–20 Years	30	307			
	>20 Years	19	252			
The location of your escapeway maps	<1 year	85	385	22.7	5	0.000
	1–5 Years	213	358			
	6–10 Years	254	343			
	11–15 Years	85	311			
	16–20 Years	30	240			
	>20 Years	19	305			
Where to report in the event of a mine emergency	<1 year	84	374	14.0	5	0.016
	1–5 Years	214	342			
	6–10 Years	253	341			
	11–15 Years	83	353			
	16–20 Years	30	241			
	>20 Years	19	329			
What the lifeline symbols mean	<1 year	88	387	54.1	5	0.000
	1–5 Years	212	395			
	6–10 Years	255	331			
	11–15 Years	85	288			
	16–20 Years	30	231			
	>20 Years	19	212			
How to reestablish ventilation	<1 year	88	396	18.1	5	0.003
	1–5 Years	211	355			
	6–10 Years	252	335			
	11–15 Years	84	289			
	16–20 Years	28	287			
	>20 Years	19	337			
Your mine’s emergency response plan (ERP)	<1 year	85	365	16.9	5	0.005
	1–5 Years	212	354			
	6–10 Years	253	335			
	11–15 Years	85	366			
	16–20 Years	30	217			
	>20 Years	19	302			

**Table 5.** Survey scale median and range by industry experience level.

Experience	% Sample	Compliance Median	Compliance Range	Risk Avoidance Median	Risk Avoidance Range
Under 1 Year	9%	5.75	3	5.50	3
1–5 Years	18%	5.50	5	5.50	3
6–10 Years	17%	5.25	5	5.50	5
11–15 Years	15%	5.50	5	5.50	5
16–20 Years	10%	5.50	5	5.50	4
20+ Years	30%	5.50	5	5.50	5

**Table 6.** Kruskal-Wallis results for mining experience and compliance.

Scale	Experience	*N*	Mean Rank	Chi-Square	df	Asymp.Sig
Compliance	0–1 year	234	1469	32.0	5	0.000
	1–5 Years	465	1320			
	6–10 Years	441	1174			
	11–15 Years	389	1238			
	16–20 Years	239	1191			
	20+ Years	761	1259			

## Appendix A. Case Study 1 Mann-Whitney Tests

**Table A1.** Mann-Whitney test results for time in position and hazard recognition accuracy.

Time in Position	*N*	Rank Average	Sum or Ranks	U	Z	*P*
Under 1 year	23	16.4	376	283	−2.67	0.004
11–20 years	4	23.8	95.0			

## Appendix B. Case Study 2 Mann-Whitney Tests

**Table A2.** Mann-Whitney results for mining experience groups and confidence in one’s ability to correctly explain or demonstrate “what to expect when wearing an SCSR”.

Time at Current Mine	*N*	Rank Average	Sum of Ranks	U	Z	*P*
0–1 Year	88	168	14,788	7784	−2.75	0.006
1–5 Years	212	143	30,362			
0–1 Year	88	199	17,536	8733	−3.56	0.000
6–10 Years	254	162	41,118			
0–1 Year	88	97.6	8586	2811	−3.41	0.001
11–15 Years	85	76.1	6466			
0–1 Year	88	64.0	5635	922	−3.12	0.002
16–20 Years	30	46.2	1387			
0–1 Year	88	58.3	5132	457	−3.91	0.000
>20 Years	19	34.0	647			

**Table A3.** Mann-Whitney results for mining experience and confidence in one’s ability to correctly explain or demonstrate “where escapeway maps are located”.

Time at Current Mine	*N*	Rank Average	Sum of Ranks	U	Z	*P*
0–1 Year	85	94.7	8047	2834	−2.92	0.004
11–15 Years	85	76.3	6489			
0–1 Year	85	75.8	6439	1382	−3.86	0.000
16–20 Years	49	53.2	2607			
1–5 Years	213	127	27,078	2104	−3.60	0.000
16–20 Years	30	85.6	2569			
6–10 Years	254	147	37,371	2634	−3.19	0.001
16–20 Years	30	103	3099			

**Table A4.** Mann-Whitney results for mining experience and confidence in one’s ability to correctly explain or demonstrate “where your RA(s) is/are located”.

Time at Current Mine	*N*	Rank Average	Sum of Ranks	U	Z	*P*
0–1 Year	85	62.0	5269	852	−2.84	0.004
16–20 Years	29	44.4	1289			
1–5 Years	213	125	26,616	2352	−2.39	0.017
16–20 Years	29	96.1	2787			

**Table A5.** Mann-Whitney results for mining experience and confidence in one’s ability to correctly explain or demonstrate “where to report in the event of a mine emergency”.

Time at Current Mine	*N*	Rank Average	Sum of Ranks	U	Z	*P*
0–1 Year	84	63.0	5291	800	−3.48	0.000
16–20 Years	30	42.2	1265			
1–5 Years	214	127	27,144	2281	−2.92	0.004
16–20 Years	30	91.5	2746			
6–10 Years	253	147	37,027	2649	−3.05	0.002
16–20 Years	30	104	3114			
11–15 Years	83	62.1	5156	821	−3.08	0.002
16–20 Years	30	42.9	1286			

**Table A6.** Mann-Whitney results for mining experience and confidence in one’s ability to correctly explain or demonstrate “what lifeline symbols mean”.

Time at Current Mine	*N*	Rank Average	Sum of Ranks	U	Z	*P*
0–1 Year	88	193	16,953	9403	−2.51	0.012
6–10 Years	255	165	42,043			
0–1 Year	88	99.2	8727	2670	−3.58	0.000
11–15 Years	85	74.4	6325			
0–1 Year	88	66.0	5808	748	−3.99	0.000
16–20 Years	30	40.4	1213			
0–1 Year	88	58.8	5177	411	−3.92	0.000
>20 Years	19	31.6	601			
1–5 Years	212	258	54,694	21,944	−3.98	0.000
6–10 Years	255	214	54,584			
1–5 Years	212	162	34,379	6220	−4.79	0.000
11–15 Years	85	116	9875			
1–5 Years	212	128	27,211	1728	−4.78	0.000
16–20 Years	30	73.1	2193			
1–5 Years	212	121	25,691	916	−4.67	0.000
>20 Years	19	58.2	1106			
6–10 Years	255	148	37,635	2655	−2.94	0.003
16–20 Years	30	104	3120			
6–10 Years	255	141	35,909	1577	−2.73	0.006
>20 Years	19	93.0	1767			

**Table A7.** Mann-Whitney results for mining experience and confidence in one’s ability to correctly explain or demonstrate “how to reestablish ventilation”.

Time at Current Mine	*N*	Rank Average	Sum of Ranks	U	Z	*P*
0–1 Year	88	193	17,020	9073	−2.69	0.007
6–10 Years	252	163	40,951			
0–1 Year	88	100	2519	2519	−3.81	0.000
11–15 Years	84	72.5	6089			
0–1 Year	88	63.0	5541	840	−2.74	0.006
16–20 Years	28	44.5	1246			
1–5 Years	211	156	32,930	7160	−2.71	0.007
11–15 Years	84	128	10,730			

**Table A8.** Mann-Whitney results for mining experience and confidence in one’s ability to correctly explain or demonstrate “how to read mine map symbols”.

Time at Current Mine	*N*	Rank Average	Sum of Ranks	U	Z	*P*
0–1 Year	88	95.5	8403	2993	−2.40	0.016
11–15 Years	85	78.2	6648			
0–1 Year	88	64.3	5655	902	−2.75	0.006
16–20 Years	30	45.6	1367			
6–10 Years	255	147	37,449	2841	−2.43	0.015
16–20 Years	30	110	3306			

## Appendix C. Case Study 3 Mann-Whitney Tests

**Table A9.** Mann-Whitney test results for mining experience groups and compliance.

Experience Group	*N*	Rank Average	Sum of Ranks	U	Z	*P*
Under 1 year	234	378	88,462	47,843	−2.68	0.007
1–5 years	465	336	156,188			
Under 1 year	234	389	90,980	39,709	−5.04	0.000
6–10 years	441	311	137,170			
Under 1 year	234	347	81,274	37,247	−3.90	0.000
11–15 years	389	291	113,102			
Under 1 year	234	263	61,532	21,889	−4.19	0.000
16–20 years	239	212	50,569			
Under 1 year	234	562	131,399	74,170	−3.96	0.000
20+ years	761	478	364,111			
1–5 years	465	479	222,852	90,559	−3.10	0.002
6–10 years	441	426	188,020			
